# Trends and Characteristics of Buprenorphine-Involved Overdose Deaths Prior to and During the COVID-19 Pandemic

**DOI:** 10.1001/jamanetworkopen.2022.51856

**Published:** 2023-01-20

**Authors:** Lauren J. Tanz, Christopher M. Jones, Nicole L. Davis, Wilson M. Compton, Grant T. Baldwin, Beth Han, Nora D. Volkow

**Affiliations:** 1 National Center for Injury Prevention and Control, Centers for Disease Control and Prevention, Atlanta, Georgia; 2 National Institute on Drug Abuse, National Institutes of Health, Bethesda, Maryland

## Abstract

**Importance:**

Buprenorphine remains underused in treating opioid use disorder, despite its effectiveness. During the onset of the COVID-19 pandemic, the US government implemented prescribing flexibilities to support continued access.

**Objective:**

To determine whether buprenorphine-involved overdose deaths changed after implementing these policy changes and highlight characteristics and circumstances of these deaths.

**Design, Setting, and Participants:**

This cross-sectional study used data from the State Unintentional Drug Overdose Reporting System (SUDORS) to assess overdose deaths in 46 states and the District of Columbia occurring July 2019 to June 2021. Data were analyzed from March 7, 2022, to June 30, 2022.

**Main Outcomes and Measures:**

Buprenorphine-involved and other opioid-involved overdose deaths were examined. Monthly opioid-involved overdose deaths and the percentage involving buprenorphine were computed to assess trends. Proportions and exact 95% CIs of drug coinvolvement, demographics, and circumstances were calculated by group.

**Results:**

During July 2019 to June 2021, 32 jurisdictions reported 89 111 total overdose deaths and 74 474 opioid-involved overdose deaths, including 1955 buprenorphine-involved overdose deaths, accounting for 2.2% of all drug overdose deaths and 2.6% of opioid-involved overdose deaths. Median (IQR) age was similar for buprenorphine-involved overdose deaths (41 [34-55] years) and other opioid–involved overdose deaths (40 [31-52] years). A higher proportion of buprenorphine-involved overdose decedents, compared with other opioid–involved decedents, were female (36.1% [95% CI, 34.2%-38.2%] vs 29.1% [95% CI, 28.8%-29.4%]), non-Hispanic White (86.1% [95% CI, 84.6%-87.6%] vs 69.4% [95% CI, 69.1%-69.7%]), and residing in rural areas (20.8% [95% CI, 19.1%-22.5%] vs 11.4% [95% CI, 11.2%-11.7%]). Although monthly opioid-involved overdose deaths increased, the proportion involving buprenorphine fluctuated but did not increase during July 2019 to June 2021. Nearly all (92.7% [95% CI, 91.5%-93.7%]) buprenorphine-involved overdose deaths involved at least 1 other drug; higher proportions involved other prescription medications compared with other opioid-involved overdose deaths (eg, anticonvulsants: 18.6% [95% CI, 17.0%-20.3%] vs 5.4% [95% CI, 5.2%-5.5%]) and a lower proportion involved illicitly manufactured fentanyls (50.2% [95% CI, 48.1%-52.3%] vs 85.3% [95% CI, 85.1%-85.5%]). Buprenorphine decedents were more likely to be receiving mental health treatment than other opioid–involved overdose decedents (31.4% [95% CI, 29.3%-33.5%] vs 13.3% [95% CI, 13.1%-13.6%]).

**Conclusions and Relevance:**

The findings of this cross-sectional study suggest that actions to facilitate access to buprenorphine-based treatment for opioid use disorder during the COVID-19 pandemic were not associated with an increased proportion of overdose deaths involving buprenorphine. Efforts are needed to expand more equitable and culturally competent access to and provision of buprenorphine-based treatment.

## Key Points

**Question:**

Did buprenorphine-involved overdose deaths change after implementing prescribing flexibilities during the COVID-19 pandemic?

**Findings:**

In this cross-sectional study including 74 474 opioid-involved overdose deaths, buprenorphine was involved in 2.6% of opioid-involved overdose deaths during July 2019 to June 2021. Although monthly opioid-involved overdose deaths increased, the proportion involving buprenorphine fluctuated but did not increase.

**Meaning:**

These findings suggest that actions to facilitate access to buprenorphine-based treatment for opioid use disorder during the COVID-19 pandemic were not associated with an increased proportion of overdose deaths involving buprenorphine; efforts are needed to expand more equitable and culturally competent access to and provision of buprenorphine-based treatment.

## Teaser

This cross-sectional study assesses whether buprenorphine-involved overdose deaths changed after implementing more flexible prescription policies during the COVID-19 pandemic and highlights characteristics and circumstances of these deaths

## Introduction

The overdose crisis in the US continues to escalate, likely associated with the widespread availability of highly potent synthetic opioids, such as illicitly manufactured fentanyl and fentanyl analogs (IMFs) in the illicit drug supply.^1,2^ Provisional data from the Centers for Disease Control and Prevention (CDC) estimate more than 107 000 overdose deaths in the US in the 12 months ending July 2022, with more than 81 000 deaths involving opioids.^3^ Expanding access to medications for opioid use disorder (OUD) is a central component of the US response to the overdose crisis.^4^

Buprenorphine is a partial mu-opioid receptor agonist with lower potential for misuse and overdose compared with the full mu-opioid receptor agonist methadone.^5^ Despite buprenorphine being the most accessible form of medication for OUD in the US, under current federal law, it can only be prescribed in office-based settings by clinicians with a Drug Addiction Treatment Act waiver; clinicians are limited to prescribing up to 30, 100, or 275 patients at a given time, depending on waiver limit.^6,7^ Therapeutic benefits of buprenorphine treatment include reduced illicit opioid use and prescription opioid misuse, decreased risk for injection-related infectious diseases, and decreased risk for fatal and nonfatal overdoses.^5,8,9,10,11,12,13,14^ Yet, buprenorphine treatment remains substantially underused.^5^

During the emergence of the COVID-19 pandemic, there were concerns for increased overdose risk among individuals with OUD from disruption to medications for OUD and other treatment access due to stay-at-home orders and temporary closures of medical and social services.^15,16^ To facilitate continued access to care for individuals with OUD, the US federal government took actions following the declaration of the nationwide emergency on March 13, 2020.^17,18^ In particular, on March 31, 2020, the Substance Abuse and Mental Health Services Administration and the Drug Enforcement Administration allowed Drug Addiction Treatment Act–waivered clinicians to remotely prescribe buprenorphine to new patients without conducting in-person examinations.^19^ On March 27, 2020, the Centers for Medicare & Medicaid Services expanded payment for telehealth services and provided flexibility on accepted communication technologies (eg, audio-only) for clinical care of substance use disorders (SUD).^20,21^

Recent studies have reported that clinicians have used these emergency authorizations to initiate and continue buprenorphine treatment during the COVID-19 pandemic and that patients have benefited.^22,23,24^ However, questions remain about whether there was an increase in buprenorphine-involved overdose deaths following implementation of these new emergency authorizations that removed historical measures intended to reduce diversion and misuse of buprenorphine.

This study assessed trends in buprenorphine-involved overdose deaths before and during the period of COVID-19–related buprenorphine prescribing flexibilities. Additionally, given very limited research on characteristics and circumstances of buprenorphine-involved overdose deaths, this study examined differences in characteristics and circumstances between buprenorphine- and other opioid–involved overdose decedents. These findings could inform ongoing policy discussions about potential permanent adoption of COVID-19 emergency authorizations related to buprenorphine prescribing and inform strategies to prevent buprenorphine-involved overdose deaths.

## Methods

This cross-sectional study was reviewed by the CDC and was deemed not to be human research under 45 CFR 46.102(l); therefore institutional review board oversight and informed consent were not required. This study was conducted consistent with applicable federal law and CDC policy. This study follows the Strengthening the Reporting of Observational Studies in Epidemiology (STROBE) reporting guideline.

### Data Source

The CDC’s State Unintentional Drug Overdose Reporting System captures information on unintentional and undetermined intent drug overdose deaths from 47 states and the District of Columbia.^25^ Jurisdictions abstract data from death certificates and medical examiner or coroner reports, including death scene investigations and postmortem toxicological findings. These sources capture drugs involved, decedent demographics, and overdose-specific circumstances.

### Trend Analysis

Trend analyses included 32 jurisdictions (31 states and the District of Columbia; eTable 1 in Supplement 1) that reported unintentional and undetermined intent drug overdose deaths that occurred during July 2019 to June 2021, the 9 months before and 15 months after COVID-19 buprenorphine prescribing flexibilities were implemented. Twenty-nine jurisdictions reported all overdose deaths in their jurisdiction and 3 jurisdictions reported deaths from subsets of counties covering at least 75% of overdose deaths in the jurisdiction. Overdose deaths were restricted to those involving (ie, listed as a cause of death) at least 1 opioid, classified by whether buprenorphine was involved, and grouped by month using death date.

### Analyses of Drug Coinvolvement, Demographics, and Urbanicity

Analyses of drug coinvolvement, decedent demographics, and urbanicity included 47 jurisdictions with death certificate data available for at least one 6-month period during July 2019 to June 2021 (eTable 1 in Supplement 1). Among these, 10 jurisdictions reported deaths from counties that accounted for at least 75% of drug overdose deaths in the state for at least one 6-month period; all other jurisdictions reported deaths from the full jurisdiction. Overdose deaths were categorized into 2 mutually exclusive groups: buprenorphine-involved and other opioid–involved. To evaluate coinvolvement of other drugs, we classified deaths into the following nonmutually exclusive groups: any other drug, any other opioid, IMFs (includes fentanyl and fentanyl analogs classified using toxicological, scene, and witness evidence^26^), cocaine, methamphetamine, prescription stimulants, benzodiazepines, antidepressants, anticonvulsants, cannabis, and alcohol. Additionally, sex, age, race and ethnicity, education, and county of residence^27^ of decedents were examined. These variables were available from the death certificate and supplemented with information from medical examiner or coroner reports. Race and ethnicity were classified as American Indian or Alaska Native, non-Hispanic; Asian or other Pacific Islander, non-Hispanic; Black, non-Hispanic; Hispanic; multiple races, non-Hispanic; and White, non-Hispanic. Race and ethnicity data were included in analyses because proportions of overdose deaths and access to treatment for OUD often vary by race and ethnicity.

### Analyses of Overdose-Specific Circumstances

Circumstance analyses were restricted to 42 jurisdictions with medical examiner or coroner reports for at least 75% of decedents, as circumstance data come primarily from these reports, and to deaths with an available medical examiner or coroner report (eTable 1 in Supplement 1). Circumstances included events of the overdose (eg, naloxone administration, potential bystander presence); scene evidence (eg, route of drug use), evidence of history of drug use and treatment (eg, current treatment for SUD), and evidence of other circumstances (eg, homelessness or housing instability).

### Statistical Analysis

Monthly opioid-involved overdose deaths and percentages of opioid-involved overdose deaths involving buprenorphine during July 2019 to June 2021 were computed. Descriptive analyses of drug coinvolvement, demographics, urbanicity, and circumstances were categorized by buprenorphine or other opioid involvement and reported as proportions and exact 95% CI for categorical variables or medians and IQRs for continuous variables. Complete case analysis was conducted and supported given limited missing data (<2% for 14 of 16 variables with missing data; <5% for 2 of 16 variables). Eleven circumstance variables were completed as checkboxes within the State Unintentional Drug Overdose Reporting System; lack of endorsement was considered lack of evidence of the circumstance and included in the denominator of proportion calculations.

Sensitivity analyses were conducted to examine whether inclusion of jurisdictions with less than 100% of death certificates (for trend, drug coinvolvement, demographics, and urbanicity analyses) or less than 90% of medical examiner or coroner reports (for circumstance analyses) changed conclusions. Additionally, to assess whether results differed before and during the COVID-19 pandemic, analyses were stratified into prepandemic (July 2019 to March 2020) and during COVID-19 (April 2020 to June 2021) time periods.

Analyses were conducted in SAS statistical software version 9.4 (SAS Institute). Data were analyzed from March 7, 2022, to June 30, 2022.

## Results

### Trend Analysis

During July 2019 to June 2021, 32 jurisdictions reported 89 111 total overdose deaths and 74 474 opioid-involved overdose deaths, including 1955 buprenorphine-involved overdose deaths, accounting for 2.2% of all drug overdose deaths and 2.6% of opioid-involved overdose deaths. Although monthly opioid-involved overdose deaths increased starting in March 2020, corresponding with the COVID-19 pandemic, the proportion with buprenorphine-involvement fluctuated but did not increase between July 2019 (3.6%) and June 2021 (2.1%) (Figure; eTable 2 in Supplement 1). Median (IQR) monthly opioid-involved overdose deaths increased 35.7% from 2520 (2468-2633) deaths during July 2019 to March 2020 to 3419 (3054-3828) deaths during April 2020 to June 2021, an increase of approximately 899 deaths per month. Median (IQR) monthly buprenorphine-involved overdose deaths increased 26.9% from 67 (65-78) deaths to 85 (80-97) deaths during the same timeframe. Nearly all of the increase in median monthly buprenorphine-involved overdose deaths was in deaths that coinvolved IMFs, which increased from a median (IQR) of 31 (28-34) deaths per month during July 2019 to March 2020 to 45 (42-52) deaths per month during April 2020 to June 2021. In sensitivity analyses, excluding jurisdictions with less than 100% of death certificates did not meaningfully change results.

**Figure.**
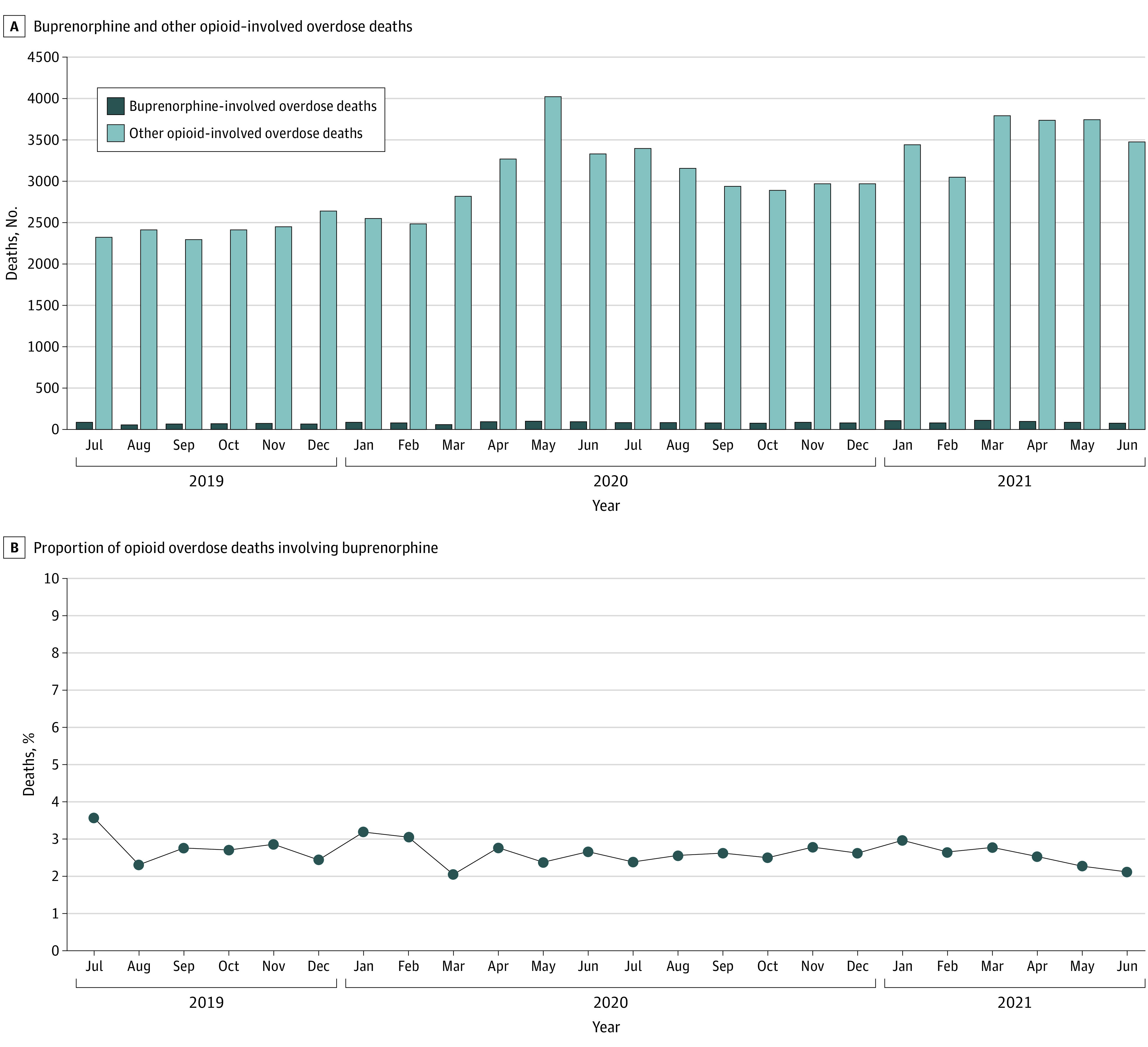
Buprenorphine- and Other Opioid–Involved Overdose Deaths in 32 US Jurisdictions From July 2019 to June 2021 Other opioid–involved overdose deaths were opioid-involved deaths that did not involve buprenorphine. Thus, the buprenorphine-involved and other opioid-involved categories are mutually exclusive and together make up all opioid-involved overdose deaths. If date of death was missing, date pronounced dead was used. The 32 included jurisdictions were Alaska, Arizona, Colorado, Connecticut, Delaware, District of Columbia, Georgia, Illinois, Kansas, Kentucky, Maine, Massachusetts, Minnesota, Missouri, Montana, Nevada, New Hampshire, New Jersey, New Mexico, North Carolina, Ohio, Oklahoma, Oregon, Pennsylvania, Rhode Island, South Dakota, Tennessee, Utah, Vermont, Virginia, Washington, and West Virginia. Illinois, Missouri, and Washington reported deaths from counties that accounted for at least 75% of drug overdose deaths in the state in 2017, per the State Unintentional Drug Overdose Reporting System funding requirements; all other jurisdictions reported deaths from the full jurisdiction.

### Analyses of Drug Coinvolvement, Demographics, and Urbanicity

Among 2238 buprenorphine-involved overdose deaths reported by 47 jurisdictions during July 2019 to June 2021, 2202 (98.4%) were categorized as unintentional and 36 (1.6%) were categorized as undetermined intent. Similarly, among 93 128 other opioid–involved overdose deaths that did not involve buprenorphine, 89 205 (95.8%) were categorized as unintentional and 3923 (4.2%) were categorized as undetermined intent. Among buprenorphine-involved overdose deaths, 92.7% (95% CI, 91.5%-93.7%) involved at least 1 other drug; only 67.2% (95% CI, 66.9%-67.5%) of other opioid–involved overdose deaths involved another drug (Table 1). The proportion of deaths involving IMFs was lower among buprenorphine-involved overdose deaths (50.2% [95% CI, 48.1%-52.3%]) compared with other opioid–involved overdose deaths (85.3% [95% CI, 85.1%-85.5%]). However, a higher proportion of buprenorphine-involved overdose deaths, compared with other opioid–involved deaths, coinvolved prescription stimulants (4.5% [95% CI, 3.7%-5.5%] vs 1.7% [95% CI, 1.6%-1.8%]), benzodiazepines (36.9% [95% CI, 34.9%-39.0%] vs 14.5% [95% CI, 14.3%-14.8%]), antidepressants (13.9% [95% CI, 12.5%-15.5%] vs 5.0% [95% CI, 4.8%-5.1%]), and anticonvulsants, primarily gabapentin and pregabalin (18.6% [95% CI, 17.0%-20.3%] vs 5.4% [95% CI, 5.2%-5.5%]).

**Table 1.** Drugs Involved, Decedent Demographic Characteristics, and Urbanicity by Buprenorphine and Other Opioid Involvement in 47 Jurisdictions From July 2019 to June 2021^a^

Characteristic	Overdose deaths, No. (%) [95% CI]	
	Buprenorphine-involved (n = 2238)	Other opioid–involved (n = 93 128)^b^
Drugs involved		
Any other drug	2074 (92.7) [91.5-93.7]	62 614 (67.2) [66.9-67.5]
Other opioid		
Any	1334 (59.6) [57.5-61.7]	93 128 (100.0) [NA]
Illicitly manufactured fentanyls	1123 (50.2) [48.1-52.3]	79 438 (85.3) [85.1-85.5]
Cocaine	474 (21.2) [19.5-22.9]	23 698 (25.4) [25.2-25.7]
Methamphetamine	478 (21.4) [19.7-23.1]	17 617 (18.9) [18.7-19.2]
Prescription stimulants	101 (4.5) [3.7-5.5]	1570 (1.7) [1.6-1.8]
Benzodiazepines	826 (36.9) [34.9-39.0]	13 522 (14.5) [14.3-14.8]
Antidepressants	312 (13.9) [12.5-15.5]	4628 (5.0) [4.8-5.1]
Anticonvulsants	416 (18.6) [17.0-20.3]	5003 (5.4) [5.2-5.5]
Cannabis	57 (2.5) [1.9-3.3]	1058 (1.1) [1.1-1.2]
Alcohol	355 (15.9) [14.4-17.4]	15 925 (17.1) [16.9-17.3]
Sex		
Male	1429 (63.9) [61.8-65.9]	66 060 (70.9) [70.6-71.2]
Female	809 (36.1) [34.2-38.2]	27 066 (29.1) [28.8-29.4]
Missing or unknown^c^	0	2
Age, y		
Median (IQR)	41 (34-51)	40 (31-52)
<18	4 (0.2) [0.1-0.5]	540 (0.6) [0.5-0.6]
18-24	87 (3.9) [3.1-4.8]	7031 (7.6) [7.4-7.7]
25-34	506 (22.6) [20.9-24.4]	24 594 (26.4) [26.1-26.7]
35-44	736 (32.9) [30.9-34.9]	24 379 (26.2) [25.9-26.5]
45-54	486 (21.7) [20.0-23.5]	18 641 (20.0) [19.8-20.3]
55-64	358 (16.0) [14.5-17.6]	14 500 (15.6) [15.3-15.8]
≥65	61 (2.7) [2.1-3.5]	3436 (3.7) [3.6-3.8]
Missing or unknown^c^	0	7
Race and ethnicity		
American Indian or Alaska Native, non-Hispanic	32 (1.4) [1.0-2.0]	981 (1.1) [1.0-1.1]
Asian or other Pacific Islander, non-Hispanic	9 (0.4) [0.2-0.8]	549 (0.6) [0.6-0.7]
Black, non-Hispanic	127 (5.7) [4.8-6.8]	17 332 (18.8) [18.5-19.0]
Hispanic	122 (5.5) [4.6-6.5]	8662 (9.4) [9.2-9.6]
Multiple races, non-Hispanic	18 (0.8) [0.5-1.3]	744 (0.8) [0.8-0.9]
White, non-Hispanic	1915 (86.1) [84.6-87.6]	64 113 (69.4) [69.1-69.7]
Missing or unknown^c^	15	747
Education		
<High school degree	479 (21.9) [20.2-23.7]	18 664 (20.7) [20.5-21.0]
High school degree or GED	1175 (53.8) [51.7-55.9]	47 888 (53.1) [52.8-53.5]
Some college or Associate’s degree	417 (19.1) [17.5-20.8]	18 127 (20.1) [19.9-20.4]
≥Bachelor’s degree	113 (5.2) [4.3-6.2]	5430 (6.0) [5.9-6.2]
Urbanicity of decedent’s county of residence^d^		
Large central metropolitan	400 (18.2) [16.6-19.9]	27 523 (30.6) [30.3-30.9]
Large fringe metropolitan	640 (29.2) [27.3-31.1]	24 519 (27.2) [27.0-27.5]
Medium metropolitan	459 (20.9) [19.2-22.7]	20 672 (23.0) [22.7-23.2]
Small metropolitan	238 (10.9) [9.6-12.2]	6990 (7.8) [7.6-7.9]
Micropolitan	267 (12.2) [10.8-13.6]	6704 (7.4) [7.3-7.6]
Noncore	189 (8.6) [7.5-9.9]	3593 (4.0) [3.9-4.1]
Missing or unknown^c^	45	3127

Abbreviation: NA, not applicable.

^a^ A list of jurisdictions is presented in eTable 1 in Supplement 1.

^b^ Includes opioid-involved overdose deaths that did not involve buprenorphine. Thus, the buprenorphine-involved and other opioid–involved categories are mutually exclusive.

^c^ Missing values were excluded from calculations of percentages. Percentages might not sum to 100% because of rounding.

^d^ Based on the 2013 NCHS Urban-Rural Classification Scheme for Counties.

A larger proportion of buprenorphine-involved overdose decedents were female compared with other opioid–involved overdose decedents (36.1% [95% CI, 34.2%-38.2%] vs 29.1% [95% CI, 28.8%-29.4%]); this was opposite for males (63.9% [95% CI, 61.8%-65.9%] vs 70.9% [95% CI, 70.6%-71.2%]) (Table 1). Although median age at death was similar across groups, a higher proportion of buprenorphine-involved deaths, compared with other opioid–involved overdose deaths, occurred in the 35 to 44 years age group and a lower proportion occurred in the 18 to 35 years age groups. Additionally, 86.1% (95% CI, 84.6%-87.6%) of buprenorphine-involved overdose deaths occurred among White, non-Hispanic persons, significantly higher than the proportion for other opioid–involved overdose deaths (69.4% [95% CI, 69.1%-69.7%]). In contrast, lower proportions of buprenorphine overdose deaths occurred in Black, non-Hispanic (5.7% [95% CI, 4.8%-6.8%]) and Hispanic (5.5% [95% CI, 4.6%-6.5%]) persons compared with proportions of other opioid–involved overdose deaths (Black, non-Hispanic: 18.8% [95% CI, 18.5%-19.0%]; Hispanic: 9.4% [95% CI, 9.2%-9.6%]). The highest proportion of overdose decedents overall had a high school degree or equivalent, but no differences in education level were identified between buprenorphine-involved and other opioid–involved overdose deaths. A lower proportion of buprenorphine-involved overdose deaths occurred in decedents living in large central metropolitan areas (18.2% [95% CI, 16.6%-19.9%]) compared with other opioid–involved overdose deaths (30.6% [95% CI, 30.3%-30.9%]); a higher proportion of buprenorphine-involved overdose deaths occurred in less urban and more rural areas (Table 1).

In sensitivity analyses, excluding jurisdictions with less than 100% of death certificates did not change conclusions on drug coinvolvement, demographics, and urbanicity. Additionally, results remained similar when stratified by whether they occurred before or during the COVID-19 pandemic.

### Analyses of Overdose-Specific Circumstances

More than 96% of overdose deaths in the 42 jurisdictions included in circumstance analyses had a medical examiner or coroner report. A higher proportion of buprenorphine-involved overdose deaths than other opioid–involved deaths occurred at home (72.0% [95% CI, 69.8%-74.1%] vs 65.2% [95% CI, 64.9%-65.6%]) and had documentation of no pulse at first responder arrival (62.2% [95% CI, 60.0%-64.4%] vs 56.3% [95% CI, 55.9%-56.7%]) (Table 2). Among buprenorphine–involved and other opioid–involved deaths, proportions of whether the drug use leading to the fatal overdose was witnessed (7.0% [95% CI, 5.9%-8.3%] vs 8.7% [95% CI, 8.5%-8.9%]) and naloxone administration (23.1% [95% CI, 21.2%-25.0%] vs 21.4% [95% CI, 21.1%-21.7%]) were similarly low.

**Table 2.** Circumstances Surrounding and Scene Evidence Identified in Overdose Deaths by Buprenorphine and Other Opioid Involvement in 42 Jurisdictions From July 2019 to June 2021^a^

Circumstances	Overdose deaths, No. (%) [95% CI]	
	Buprenorphine-involved (n = 1901)	Other opioid–involved (n = 75 882)^b^
Evidence of overdose circumstances^c^		
Overdosed at home	1259 (72.0) [69.8-74.1]	45 719 (65.2) [64.9-65.6]
Fatal drug use witnessed	134 (7.0) [5.9-8.3]	6632 (8.7) [8.5-8.9]
Potential bystander present^d^	858 (45.1) [42.9-47.4]	35 301 (46.5) [46.2-46.9]
Naloxone administered	436 (23.1) [21.2-25.0]	16 139 (21.4) [21.1-21.7]
Documentation of no pulse at first responder arrival	1158 (62.2) [60.0-64.4]	42 005 (56.3) [55.9-56.7]
Seen in emergency department	338 (18.0) [16.3-19.8]	15 408 (20.5) [20.2-20.8]
Scene evidence		
Route of drug use^e^^,^^f^		
Injection	517 (27.2) [25.2-29.3]	18 511 (24.4) [24.1-24.7]
Smoking	186 (9.8) [8.5-11.2]	10 733 (14.1) [13.9-14.4]
Snorting	181 (9.5) [8.2-10.9]	11 521 (15.2) [14.9-15.4]
Ingestion	271 (14.3) [12.7-15.9]	11 304 (14.9) [14.6-15.2]
Other^g^	53 (2.8) [2.1-3.6]	369 (0.5) [0.4-0.5]
No reported route of drug use	978 (51.4) [49.2-53.7]	36 198 (47.7) [47.4-48.1]
Evidence of drugs on scene^f^		
Prescription drugs	560 (29.5) [27.4-31.6]	18 184 (24.0) [23.7-24.3]
Illicit drugs	540 (28.4) [26.4-30.5]	29 234 (38.5) [38.2-38.9]
Evidence of history of drug use and treatment		
History of opioid use	882 (46.4) [44.1-48.7]	32 088 (42.3) [41.9-42.6]
Recent return to use of opioids^c^	180 (9.5) [8.2-10.9]	5894 (7.8) [7.6-8.0]
Prior overdose in the past year^c^	107 (5.6) [4.6-6.8]	4782 (6.3) [6.1-6.5]
Current treatment for substance use disorders^c^^,^^f^^,^^h^		
Any	428 (22.5) [20.7-24.5]	4482 (5.9) [5.7-6.1]
Rehabilitation	32 (1.7) [1.2-2.4]	1488 (2.0) [1.9-2.1]
Medications for opioid use disorder	384 (20.2) [18.4-22.1]	2421 (3.2) [3.1-3.3]
Cognitive/behavioral therapy	24 (1.3) [0.8-1.9]	200 (0.3) [0.2-0.3]
Narcotics anonymous	3 (0.2) [0.03-0.5]	104 (0.1) [0.1-0.2]
Other^e^	20 (1.1) [0.6-1.6]	610 (0.8) [0.7-0.9]
Current treatment for pain^c^^,^^h^	166 (8.7) [7.5-10.1]	6261 (8.3) [8.1-8.5]
Evidence of other circumstances^c^		
Recent release from institutional setting	131 (7.0) [5.9-8.3]	6795 (9.1) [8.9-9.3]
Experiencing homelessness or housing instability^i^	107 (5.7) [4.7-6.9]	5029 (6.7) [6.5-6.9]
Mental health diagnosis	584 (30.7) [28.7-32.9]	17 395 (22.9) [22.6-23.2]
Current mental health treatment^j^	596 (31.4) [29.3-33.5]	10 121 (13.3) [13.1-13.6]

^a^ A list of jurisdictions is presented in eTable 1 in Supplement 1.

^b^ Includes opioid-involved overdose deaths that did not involve buprenorphine. Thus, the buprenorphine-involved and other opioid–involved categories are mutually exclusive.

^c^ Missing values were excluded from calculations of percentages.

^d^ A potential bystander is defined as a person 11 years or older who was physically nearby either during or shortly preceding a drug overdose and potentially had an opportunity to intervene or respond to the overdose. This includes persons in the same structure (eg, same room or same building but different room) as the decedent during that time. This does not include persons in different self-contained parts of larger buildings (eg, a person in a different apartment in the same apartment building).

^e^ Route of drug use cannot be directly linked to specific drugs if more than 1 drug was detected and more than 1 route was reported.

^f^ Categories are not mutually exclusive.

^g^ Includes transdermal, suppository, sublingual, and buccal.

^h^ Current treatment is defined as being treated at the time of the fatal overdose.

^i^ Persons experiencing homelessness were those who resided in places not designed for or ordinarily used as regular sleeping accommodations or in a supervised shelter or drop-in center designated to provide temporary living arrangements. Persons experiencing housing instability were those who lack resources to obtain or retain permanent housing and includes interrelated challenges (eg, trouble paying rent, overcrowding, moving frequently, or staying with relatives).

^j^ Current mental health treatment includes treatment for both mental health and substance use disorders at the time of the fatal overdose.

Although approximately half of decedents in each group had no reported route of drug use, a lower proportion of buprenorphine-involved overdose decedents had evidence of smoking (9.8% [95% CI, 8.5%-11.2%]) and snorting (9.5% [95% CI, 8.2%-10.9%]) compared with other opioid–involved decedents (smoking: 14.1% [95% CI, 13.9%-14.4%]; snorting: 15.2% [95% CI, 14.9%-15.4%]) (Table 2). Evidence of illicit drugs on scene was lower among buprenorphine-involved deaths (28.4% [95% CI, 26.4%-30.5%]) than other opioid–involved deaths (38.5% [95% CI, 38.2%-38.9%]).

Less than a one-fourth of buprenorphine-involved overdose decedents were reportedly receiving treatment for SUD (22.5% [95% CI, 20.7%-24.5%]), with 20.2% (95% CI, 18.4%-22.1%) of decedents specifically receiving medications for OUD (Table 2). In contrast, only 5.9% (95% CI, 5.7%-6.1%) of other opioid–involved overdose decedents were reportedly receiving treatment, with only 3.2% (95% CI, 3.1%-3.3%) receiving medications for OUD. Current SUD treatment results were similar when stratifying by urban and rural county of residence. Similarly, among buprenorphine-involved overdose deaths, 30.7% (95% CI, 28.7%-32.9%) were persons with a reported mental health diagnosis and 31.4% (95% CI, 29.3%-33.5%) were persons reportedly receiving mental health treatment. Proportions were lower among other opioid–involved overdose deaths, with 22.9% (95% CI, 22.6%-23.2%) of decedents having a reported mental health diagnosis and only 13.3% (95% CI, 13.1%-13.6%) of decedents receiving mental health treatment at the time of the fatal overdose.

In sensitivity analyses, excluding jurisdictions with medical examiner or coroner reports available for less than 90% of overdose deaths in their jurisdiction did not change conclusions. Similarly, stratifying analyses by before or during COVID-19 did not change conclusions.

## Discussion

This cross-sectional study found that buprenorphine was involved in a very small proportion of drug overdose deaths (2.2%) and opioid-involved overdose deaths (2.6%) in the US during July 2019 to June 2021. Importantly, the proportion of buprenorphine-involved overdose deaths fluctuated but did not increase during the 15 months from April 2020 to June 2021 when buprenorphine prescribing regulations were relaxed due to the COVID-19 pandemic. These findings have important policy implications when policy makers consider whether COVID-19–related buprenorphine prescribing flexibilities should be permanently adopted. Additionally, our findings are consistent with a 2022 study reporting no association between COVID-19–related prescribing flexibilities for methadone-based OUD treatment and methadone-involved overdose deaths.^28^

Our data show that median monthly buprenorphine-involved overdose deaths increased less than opioid-involved overdose deaths from before the pandemic to during the pandemic, even with expanded access. Moreover, most of the increase was deaths that coinvolved IMFs. Given continued expansion of buprenorphine prescribing—2021 data show more than 1 million patients receiving buprenorphine from retail pharmacies in the US^29^—our findings suggest that expanded prescribing was not associated with a disproportionate number of deaths involving buprenorphine.

Characteristics of overdose deaths in this analysis provide important insights about potential ways to improve safety and clinical outcomes. First, nearly all (92.7%) buprenorphine-involved overdose deaths involved at least 1 other drug, reflecting the complex nature of polysubstance use and SUD.^30^ Second, compared with other opioid–involved deaths, buprenorphine-involved overdose deaths were more likely to involve prescription medications (stimulants, benzodiazepines, antidepressants, and anticonvulsants) and less likely to involve IMFs. Buprenorphine-involved decedents were also more likely to be receiving mental health treatment and to die at home. Most overdose deaths, regardless of drugs involved, occurred without another person being present, a known risk factor for fatal overdose.^31^ Together, these findings highlight the need to advance programmatic and clinical strategies that embrace the complexity of polysubstance use rather than single-drug approaches, address cooccurring mental health and SUD in a comprehensive and coordinated manner, and integrate provision of naloxone and overdose prevention education for both individuals at risk for overdose and family members, caregivers, or others who might be in a position to respond to overdoses.

Although a larger proportion of buprenorphine-involved decedents had evidence of current treatment for SUD compared with other opioid–involved decedents, most individuals in both groups (78% and 94%, respectively) had no evidence of current treatment. This stark finding highlights the need to expand access to evidence-based treatment, particularly medications for OUD; improve treatment retention; and support long-term recovery. Furthermore, the large percentage of buprenorphine-involved overdose decedents without evidence of treatment may reflect buprenorphine misuse to suppress withdrawal and self-treat OUD in the absence of formal treatment access. Prior research has shown that motivations for buprenorphine misuse are primarily associated with treatment outcomes (eg, suppression of withdrawal) rather than related to euphoria.^32,33^ Finally, the finding that a larger proportion of buprenorphine-involved overdose deaths, compared with other opioid–involved overdose deaths, were White non-Hispanic persons, may reflect lower rates of buprenorphine treatment among Black and Hispanic individuals.^34,35^ Disproportionate increases in overdose death rates have been reported among American Indian, Alaska Native, and Black persons compared with White persons in counties with higher SUD treatment availability.^36^ This may reflect treatment access barriers, including mistrust in the health care system, stigma, transportation access, and insurance status.^36,37^ Policy and structural interventions are needed for more equitable access to medications for OUD among people from racial and ethnic minority groups, such as American Indian, Alaska Native, and Black individuals.^36,37^

### Limitations

This study has some limitations. Analyses were limited to states with data available for deaths during July 2019 to June 2021; therefore, results might not be generalizable to the entire country. Ten states submitted data on subsets of counties, which could have impacted results; however, sensitivity analyses excluding them did not yield meaningfully different results. Similarly, twelve jurisdictions did not have 100% of death certificates for drug coinvolvement, demographics, and urbanicity analyses, and 4 states had less than 90% of medical examiner or coroner reports for circumstances analyses; their exclusion did not change conclusions. Medical examiner and coroner reports also likely underestimate circumstances because death investigators may have limited information. The time-frame included in trend, drug coinvolvement, urbanicity, and circumstances analyses spanned the prepandemic and pandemic periods, and combining these timeframes may have masked differences over time. However, analyses stratified by time period did not identify significant differences. Despite these limitations, to our knowledge, this is the most extensive assessment of buprenorphine-involved overdose deaths in the US to date.

## Conclusions

The findings of this cross-sectional study suggest that actions taken by the US federal government to facilitate access to buprenorphine-based medications for OUD during the pandemic were not associated with an increased proportion of overdose deaths involving buprenorphine, providing evidence to inform discussions on permanent adoption of COVID-19–related buprenorphine prescribing authorities. Nonetheless, although rare, overdose deaths involving buprenorphine highlight the importance of overdose prevention and support for those using buprenorphine both under medical supervision or outside of treatment for SUD or pain. Efforts to expand more equitable provision of medications for OUD and harm reduction strategies are needed to address the increasing overdose crisis.

## Notes

Supplement 1. **eTable 1.** Jurisdictions Included in Each Analysis **eTable 2.** Number of Buprenorphine and Other Opioid–Involved Overdose Deaths and Percentage of Opioid Overdose Deaths Involving Buprenorphine by Month of Death in 32 Jurisdictions From July 2019 to June 2021

Supplement 2.
Data Sharing Statement
