# Supplementary material for: Trends and Characteristics of Buprenorphine-Involved Overdose Deaths Prior to and During the COVID-19 Pandemic
Source: JAMA Netw Open. 2023 Jan 20;6(1):e2251856. doi: 10.1001/jamanetworkopen.2022.51856 (PMC9860517; doi:10.1001/jamanetworkopen.2022.51856)
Supplement: Supplement 1. — eTable 1. Jurisdictions Included in Each Analysis eTable 2. Number of Buprenorphine and Other Opioid–Involved Overdose Deaths and Percentage of Opioid Overdose Deaths Involving Buprenorphine by Month of Death in 32 Jurisdictions From July 2019 to June 2021 [file jamanetwopen-e2251856-s001.pdf]

Supplemental Online Content

Tanz LJ, Jones CM, Davis NL, et al. Trends and characteristics of buprenorphine-involved overdose deaths prior to and during the COVID-19 pandemic. *JAMA Netw Open*. 2023;6(1):e2251856. doi:10.1001/jamanetworkopen.2022.51856

eTable 1. Jurisdictions Included in Each Analysis

eTable 2. Number of Buprenorphine and Other Opioid–Involved Overdose Deaths and Percentage of Opioid Overdose Deaths Involving Buprenorphine by Month of Death in 32 Jurisdictions From July 2019 to June 2021

This supplemental material has been provided by the authors to give readers additional information about their work.

© 2023 Tanz LJ et al. *JAMA Network Open.*

eTable 1. Jurisdictions Included in Each Analysis

Jurisdiction	Trends (Figure 1)[a]	Drug co-involvement, demographics, and urbanicity (Table 1)[b]	Overdose circumstances (Table 2)[c]
	N=32	N=47	N=42
Alabama		X	
Alaska	X	X	X
Arizona	X	X	X
Arkansas		X	X
Colorado	X	X	X
Connecticut	X	X	X
Delaware	X	X	X
District of Columbia	X	X	X
Florida		X	
Georgia	X	X	X
Hawaii		X	X
Idaho		X	
Illinois	X	X	X
Indiana		X	X
Iowa		X	X
Kansas	X	X	X
Kentucky	X	X	X
Louisiana		X	X
Maine	X	X	X
Maryland		X	X
Massachusetts	X	X	X
Michigan		X	X
Minnesota	X	X	X
Mississippi		X	X
Missouri	X	X	X
Montana	X	X	X
Nebraska		X	X
Nevada	X	X	X
New Hampshire	X	X	X
New Jersey	X	X	X
New Mexico	X	X	X
New York		X	
North Carolina	X	X	X
Ohio	X	X	X
Oklahoma	X	X	X
Oregon	X	X	X
Pennsylvania	X	X	X
Rhode Island	X	X	X
South Carolina		X	
South Dakota	X	X	X
Tennessee	X	X	X
Utah	X	X	X

© 2023 Tanz LJ et al. *JAMA Network Open.*

Vermont	X	X	X
Virginia	X	X	X
Washington	X	X	X
West Virginia	X	X	X
Wisconsin		X	X
"X" denotes inclusion in the specified analysis			
[a]Includes jurisdictions that reported unintentional and undetermined intent drug overdose deaths from counties representing ≥75% of overdose deaths for every 6-month period from July 2019–June 2021 (i.e., July–December 2019, January–June 2020, July–December 2020, and January–June 2021).			
[b]Includes jurisdictions with death certificate data available from counties representing ≥75% of overdose deaths for at least one six-month period during July 2019–June 2021 (i.e., July–December 2019, January–June 2020, July–December 2020, or January–June 2021).			
[c]Includes jurisdictions with death certificate data available from counties representing ≥75% of overdose deaths and medical examiner or coroner reports available for ≥75% of deaths for at least one six-month period during July 2019–June 2021 (i.e., July–December 2019, January–June 2020, July–December 2020, or January–June 2021).			

© 2023 Tanz LJ et al. *JAMA Network Open.*

eTable 2. Number of Buprenorphine and Other Opioid–Involved Overdose Deaths[a] and Percentage of Opioid Overdose Deaths Involving Buprenorphine by Month of Death[b] in 32 Jurisdictions[c] From July 2019 to June 2021				
	Buprenorphine-involved overdose deaths	Other opioid-involved overdose deaths[a]	All opioid involved overdose deaths[d]	Percent of opioid overdose deaths involving buprenorphine[e]
	N	N	N	%
July 2019	86	2,322	2,408	3.6
August 2019	57	2,411	2,468	2.3
September 2019	65	2,294	2,359	2.8
October 2019	67	2,410	2,477	2.7
November 2019	72	2,448	2,520	2.9
December 2019	66	2,637	2,703	2.4
January 2020	84	2,549	2,633	3.2
February 2020	78	2,481	2,559	3.0
March 2020	59	2,817	2,876	2.1
April 2020	93	3,268	3,361	2.8
May 2020	98	4,019	4,117	2.4
June 2020	91	3,328	3,419	2.7
July 2020	83	3,394	3,477	2.4
August 2020	83	3,155	3,238	2.6
September 2020	79	2,936	3,015	2.6
October 2020	74	2,887	2,961	2.5
November 2020	85	2,969	3,054	2.8
December 2020	80	2,969	3,049	2.6
January 2021	105	3,440	3,545	3.0
February 2021	83	3,048	3,131	2.7
March 2021	108	3,788	3,896	2.8
April 2021	97	3,735	3,832	2.5
May 2021	87	3,741	3,828	2.3
June 2021	75	3,473	3,548	2.1

[a]Includes opioid-involved overdose deaths that do not involve buprenorphine. Thus, the buprenorphine-involved and other opioid-involved categories are mutually exclusive and together make up all opioid-involved overdose deaths.

[b]Based on date of death. If date of death was missing, date pronounced dead was used.

[c]Includes Alaska, Arizona, Colorado, Connecticut, Delaware, District of Columbia, Georgia, Illinois, Kansas, Kentucky, Maine, Massachusetts, Minnesota, Missouri, Montana, Nevada, New Hampshire, New Jersey, New Mexico, North Carolina, Ohio, Oklahoma, Oregon, Pennsylvania, Rhode Island, South Dakota, Tennessee, Utah, Vermont, Virginia, Washington, and West Virginia. Illinois, Missouri, and Washington reported deaths from counties that accounted for ≥75% of drug overdose deaths in the state in 2017, per the State Unintentional Drug Overdose Reporting System funding requirements; all other jurisdictions reported deaths from the full jurisdiction.

[d]Represents the sum of buprenorphine-involved overdose deaths and other opioid-involved overdose deaths.

[e]Calculated by dividing the number of buprenorphine-involved overdose deaths by all opioid overdose deaths.

© 2023 Tanz LJ et al. *JAMA Network Open.*
