# Supplementary material for: Trends and Characteristics of Buprenorphine-Involved Overdose Deaths Prior to and During the COVID-19 Pandemic
Source: JAMA Netw Open. 2023 Jan 20;6(1):e2251856. doi: 10.1001/jamanetworkopen.2022.51856 (PMC9860517; doi:10.1001/jamanetworkopen.2022.51856)
Supplement: Supplement 2. — Data Sharing Statement [file jamanetwopen-e2251856-s002.pdf]

Data Sharing Statement

Tanz. Trends and Characteristics of Buprenorphine-Involved Overdose Deaths Prior to and During the COVID-19 Pandemic. *JAMA Netw Open*. Published January 20, 2023. doi:10.1001/jamanetworkopen.2022.51856

Data
Data available: No

Additional Information
Explanation for why data not available: The data underlying this article may not be shared due to data sharing agreements in place with state health departments.
